# Environmental Isolation of Candida auris from the Coastal Wetlands of Andaman Islands, India

**DOI:** 10.1128/mBio.03181-20

**Published:** 2021-03-16

**Authors:** Parth Arora, Prerna Singh, Yue Wang, Anamika Yadav, Kalpana Pawar, Ashutosh Singh, Gadi Padmavati, Jianping Xu, Anuradha Chowdhary

**Affiliations:** a Department of Medical Mycology, Vallabhbhai Patel Chest Institute, University of Delhi, New Delhi, India; b Department of Ocean Studies and Marine Biology, Pondicherry University, Port Blair, Andaman & Nicobar Islands, India; c Department of Biology, McMaster University, Hamilton, Ontario, Canada; University of Toronto

**Keywords:** *Candida auris*, natural habitat, marine environment, ecology, wetlands, Andaman Islands, India

## Abstract

Candida auris is a multidrug resistant pathogen that presents a serious global threat to human health. As C. auris is a newly emerged pathogen, several questions regarding its ecological niche remain unexplored. While species closely related to C. auris have been detected in different environmental habitats, little is known about the natural habitat(s) of C. auris. Here, we explored the virgin habitats around the very isolated Andaman Islands in the Indian Ocean for evidence of C. auris. We sampled coastal wetlands, including rocky shores, sandy beaches, tidal marshes, and mangrove swamps, around the Andaman group of the Andaman & Nicobar Islands, Union Territory, in India. Forty-eight samples of sediment soil and seawater were collected from eight sampling sites representing the heterogeneity of intertidal habitats across the east and west coast of South Andaman district. C. auris was isolated from two of the eight sampling sites, a salt marsh and a sandy beach. Interestingly, both multidrug-susceptible and multidrug-resistant C. auris isolates were found in the sample. Whole-genome sequencing analysis clustered the C. auris isolates into clade I, showing close similarity to other isolates from South Asia. Isolation of C. auris from the tropical coastal environment suggests its association with the marine ecosystem. The fact that viable C. auris was detected in the marine habitat confirms C. auris survival in harsh wetlands. However, the ecological significance of C. auris in salt marsh wetland and sandy beaches to human infections remains to be explored.

## Abstract (precis)

Candida auris is a recently emerged multidrug-resistant fungal pathogen capable of causing severe infections in hospitalized patients. Despite its recognition as a human pathogen a decade ago, so far the natural ecological niche(s) of C. auris remains enigmatic.

## OBSERVATION

Candida auris is a multidrug-resistant fungal pathogen that presents a serious threat to patients and health care facilities worldwide (1). Due to the widespread clinical and economic impact of difficult-to-treat C. auris infections, the U.S. Centers for Disease Control and Prevention in 2019 have classified C. auris as an urgent threat to public health (2). An escalating number of infections across the globe in health care facilities have been attributed to the unique characteristics of this yeast in that it can survive and persist within the hospital environment for prolonged periods. Its ability to survive on dry environmental surfaces for prolonged periods suggests that this yeast is well adapted to survival outside human host settings (3). Candida auris belongs to the Candida haemulonii clade of the Metschnikowiaceae family of the order Saccharomycetales. The related species of the Metschnikowiaceae family have been detected in plants, insects, and aquatic environments, as well as from human body sites (4, 5). The closest known relative of C. auris is *C. haemulonii*, which was first discovered in 1962 from the gut of a blue-striped grunt fish (Haemulon sciurus), the skin of dolphins, and the seawater off the coast of Portugal (6). In contrast to the related species, C. auris has not been reported from natural environments. The retrospective analyses of clinical yeasts showed that the earliest known clinical isolates of C. auris date back to 1996 in South Korea and 1997 in Japan (7–9). However, unlike the clinical settings, the detection of C. auris in the natural environment has not been explored (10).

Candida auris is capable of growing at higher temperatures than most of its closely related species and can tolerate hypersaline environments more than most *Candida* species (11). The thermotolerance of C. auris has led to the hypothesis by Casadevall et al. that its emergence may be linked to climate change and global temperature changes (12). Indeed, the authors proposed that prior to its recognition as a human pathogen and being prevalent in hospital environments; C. auris was an environmental fungus that might have previously existed as a plant saprophyte in specialized ecosystems, such as wetlands. Its emergence might have been linked to global warming effects on wetlands, and its enrichment in that ecological niche was the result of C. auris’s combined thermal tolerance and salinity tolerance. Wetlands are among the crucial natural habitats distributed throughout the world and contain an enormous diversity of organisms, including yeast species. Following the prediction of Casadevall’s hypothesis, we undertook the present study to explore the environmental niches of C. auris in the marine environment encompassing coastal wetlands including rocky shores, tidal marshes, and mangrove swamps of the Andaman Islands, Union Territory of India.

**FIG 1:**
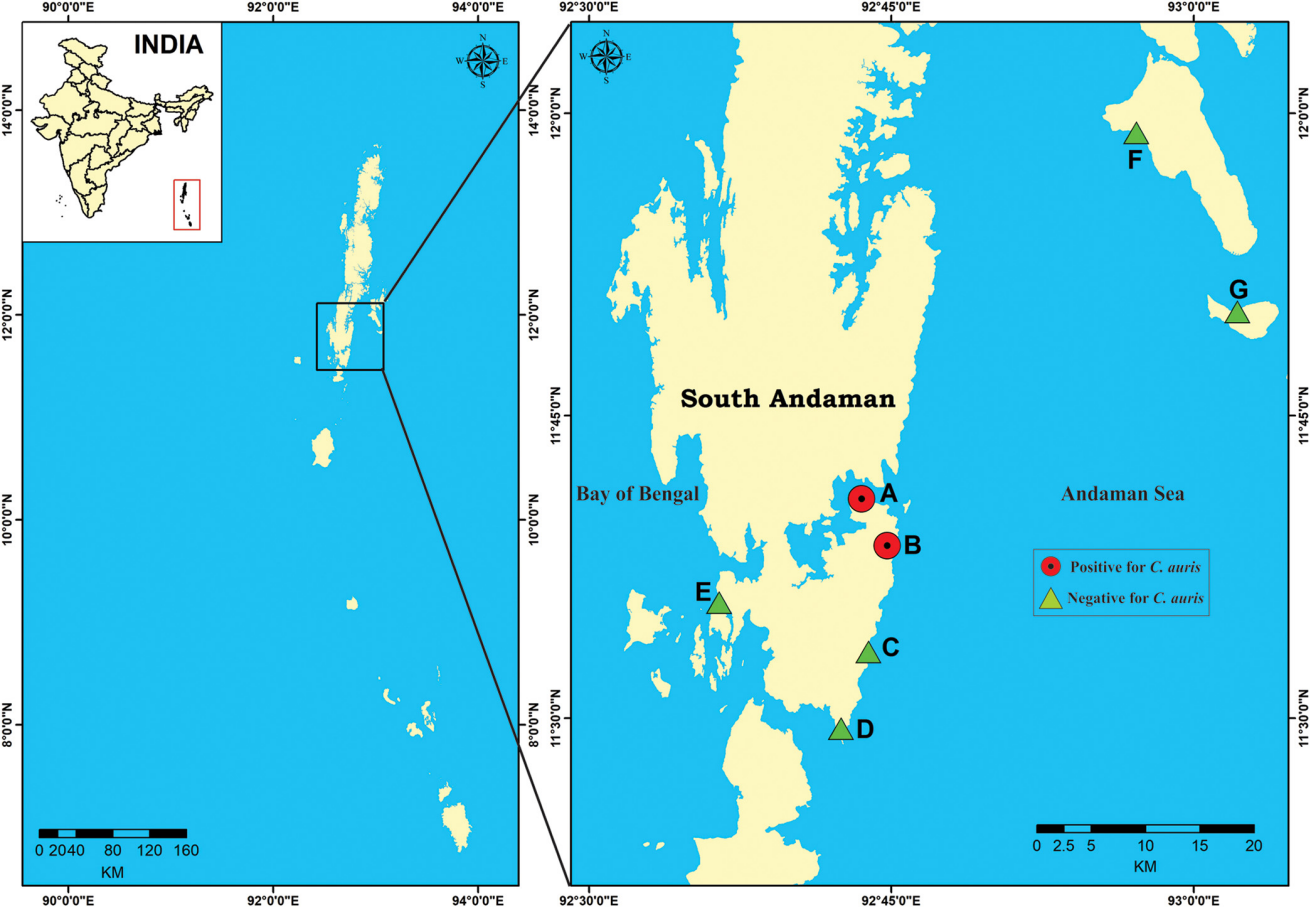
Map showing location of sampling sites (*n* = 8; sites E1 and E2 collectively depicted as E) of South Andaman district, Andaman & Nicobar Islands, Union Territory of India.

The Andaman & Nicobar Islands of the Union Territory of India are a chain of islands located in the southeastern Bay of Bengal and are surrounded by the Andaman Sea to the east and the Bay of Bengal to the west. The climate of these islands is tropical, with hot and humid conditions. In the present study, eight sampling sites including six on the east coast of South Andaman district and two on the west coast were selected to represent the heterogeneity of intertidal habitats and accessibility for specimen collections (Fig. 1). The coastal zone of the South Andaman district is endowed with sandy beaches and mangrove vegetation interspersed with rocky outcrops. Corals, seaweeds, and seagrasses are common in this region. Due to its unique location and tribal culture, few people visit these islands. Thus, we expect little impacts of direct human activities on their yeast distributions.

Candida auris was isolated from two of the eight locations investigated in the present study: a salt marsh wetland site (site A, Chatham) and a sandy beach site (site B, Corbyn’s Cove) as detailed in Table 1. Candida auris strains at site A were isolated from the sediment samples while those at site B were from both the sediment and water samples. Site A is a bay tidal salt marsh which is directly in contact with seawater of the Andaman Sea, and the marsh was exposed only during low tide. The intertidal area is separated from the terrestrial land by a seawall. The habitat is characterized by a mixture of sandy-muddy substratum and an abundant vegetation of seagrass. The area is inhabited by sea birds and experiences no known human activity. The Corbyn’s Cove beach site that yielded C. auris was an upper middle intertidal zone with the shore characterized by the presence of fine sand sediment and uprooted seaweeds. In total, two colonies of C. auris were found at site A, and both were from the composite soil sediment. In contrast, 22 colonies were found at site B, and they were distributed in both the sediment and water samples. Further, both sites A and B had low yeast diversity with only C. auris and Trichosporon asahii cultured from site A samples and C. auris and Candida parapsilosis from site B samples.

**TABLE 1:** Description of study area locations and distribution of yeast species isolated from the South Andaman district (SAD), Andaman & Nicobar Islands, Union Territory of India

Sampling location	Sampling station	Yeast species isolated (no. of colonies isolated)	Station description
Location I (South Andaman Island)	A (Chatham salt marsh)	**Candida auris** (*n*** = 2**)^*a*^ Trichosporon asahii (*n* = 4) Arthrographis kalrae^*c*^ (*n* = 2)	Intertidal habitat along the east coast of SAD, characterized by marshy sediment on the abundant seagrass bed with seabirds; negligible human activity.
	B (Corbyn’s Cove)	**Candida auris** (*n*** = 22**)^*b*^ Candida parapsilosis (*n* = 2)	Upper middle intertidal zone, east coast of SAD, a tourist beach with fine sand sediment
	C (Burmanallah rocky shore)	Candida tropicalis (*n* = 5)	Rocky shore, intertidal habitat, coarse sediment, rocky substratum with dead corals
	D (Chidiyatapu)	Candida parapsilosis (*n* = 6)	Southernmost tip of SAD, surrounded by waters of Andaman Sea and Bay of Bengal, sediment comprised of granules and pebbles, coastal birds, crocodile-prone area, with fewer human activities
	E_1_ (Wandoor)	Candida parapsilosis (*n* = 10) Trichosporon asahii (*n* = 5)	West coast of SAD, crocodile-inhabited area, coastal birds, minimal human activity
	E_2_ (Wandoor mangrove)	Candida catenulata (*n* = 5) Candida tropicalis (*n* = 2)	West coast of SAD

Location II (Swaraj Dweep Island)	F (Radhanagar)	None	Tourist beach

Location III (Shaheed Dweep Island)	G (Bharatpur)	Candida parapsilosis (*n* = 11) Candida palmioleophila (*n* = 1) Issatchenkia siamensis (*n* = 1) Kluyveromyces siamensis (*n* = 1) Kluyveromyces aestuarii (*n* = 1)	Beach with water sports activities

a Number of colonies isolated from the sediment suspension. Boldface highlights the isolation of *C. auris* from site A and site B.

b Number of colonies isolated from the sediment suspension and seawater.

c Hyaline mold.

**FIG 2:**
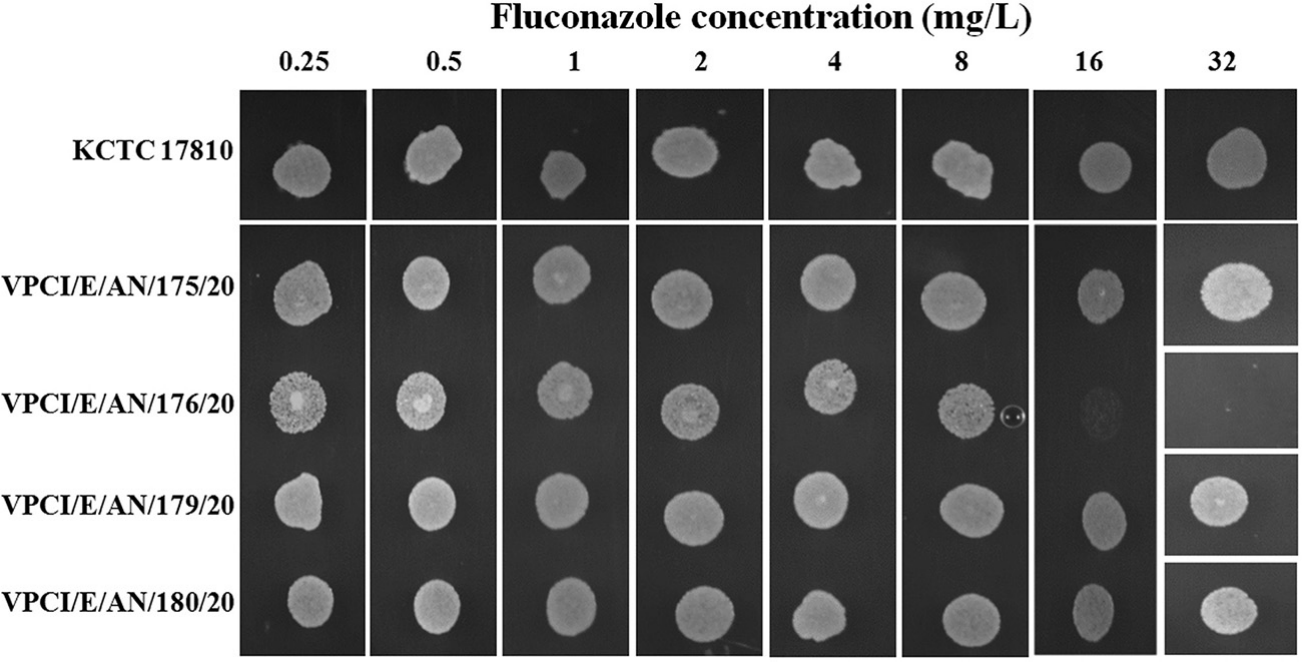
Representation of fluconazole susceptibility profile by spot assay on fluconazole-containing agar plates. Fluconazole concentrations used are depicted horizontally. Both fluconazole-susceptible (VPCI/E/AN/176/20) and -resistant (VPCI/E/AN/175/20) C. auris strains from site A and two resistant strains from site B (VPCI/E/AN/179/20 and VPCI/E/AN/180/20) of South Andaman District, Andaman & Nicobar Islands, Union Territory of India, were spot inoculated. The KCTC 17810 (clade II) strain of C. auris was used as a reference strain. Spots on the plate indicate growth of C. auris at that concentration.

**FIG 3:**
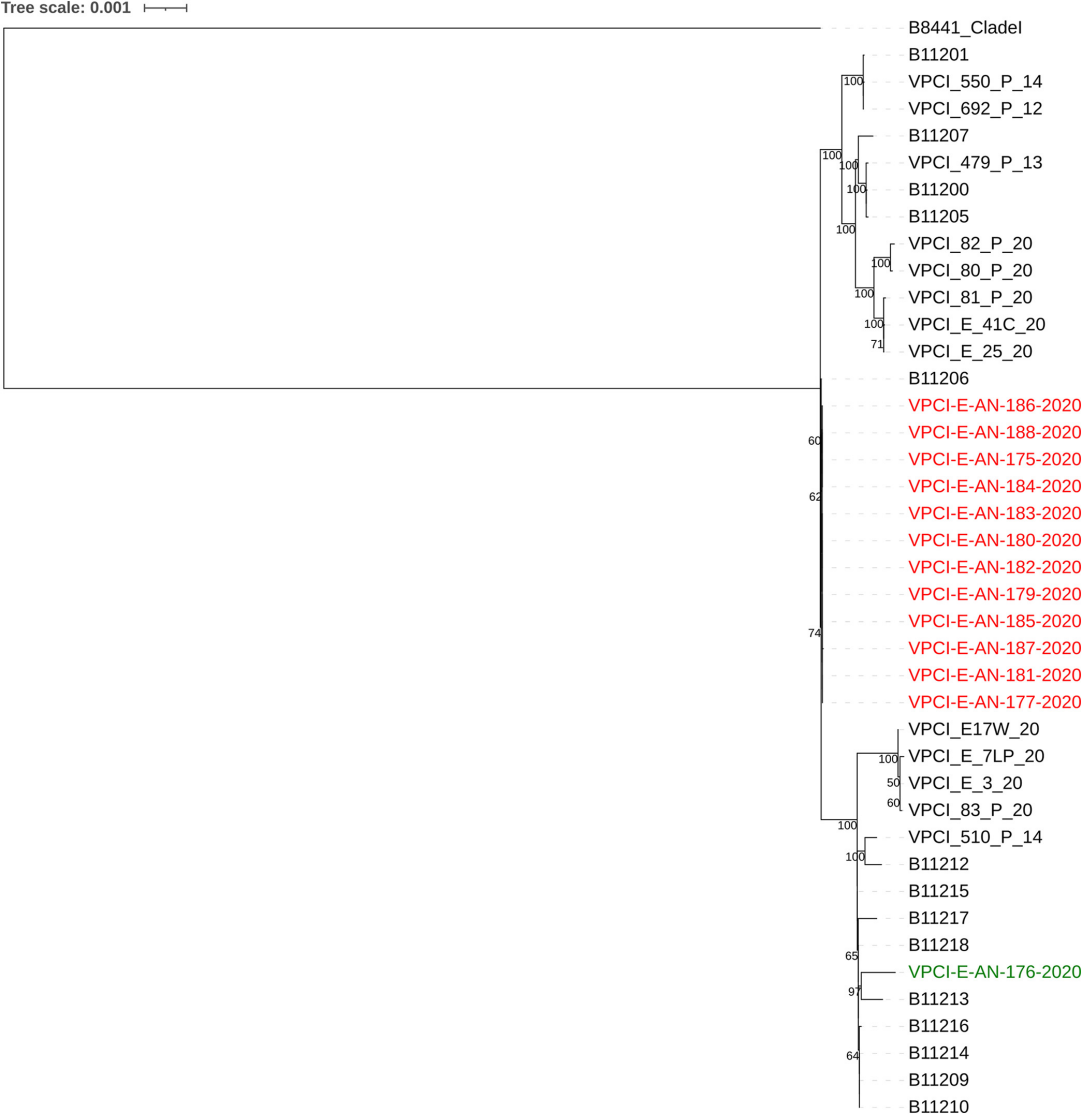
Maximum likelihood phylogenetic tree constructed based on whole-genome SNPs using RAxML. Included in the phylogenetic tree are 13 Candida auris strains isolated from South Andaman district, Andaman & Nicobar Islands (VPCI/E/AN/175/20 to AN/177/20 and VPCI/E/AN/179/20 to AN/188/20). Red text in the figure represents resistant strains (fluconazole geometric mean [GM] MIC, 256 mg/liter; amphotericin B GM MIC, 4 mg/liter) from both sites A and B, and the green text represents a single susceptible strain (fluconazole MIC, 8 mg/liter; amphotericin B MIC, 1 mg/liter) from site A. The remaining strains depicted include 9 recent isolates from patients and their immediate environments (VPCI/80/P/20 to VPCI/82/P/20, VPCI/E/41C/20, VPCI/E/25/20, VPCI/E/17W/20, VPCI/E/7LP/20, VPCI/E/3/20, and VPCI/83/P/20), 18 previously published Indian C. auris strains (B11200, B11201, B11205 to B11207, B11209, B11210, B11212 to B11218, VPCI/510/P/14, VPCI/692/P/12, VPCI/550/P/14, and VPCI/479/P/13), and reference clade I strain B8441 from Pakistan.

Antifungal susceptibility testing by the CLSI broth microdilution method showed that 23 of the 24 C. auris isolates had high MICs of fluconazole (MIC of >256 mg/liter) and amphotericin B (MIC range of 2 to 4 mg/liter) while a single isolate (VPCI/E/AN/176/20) had low MICs against all tested antifungal drugs (e.g., fluconazole MIC of 8 mg/liter and amphotericin B MIC of 1 mg/liter) (see Table S1 in the supplemental material) (13). This susceptible isolate was recovered from the salt marsh (site A). Interestingly, the other C. auris isolate from the same salt marsh exhibited high MIC values against azoles and amphotericin B. Also, all 22 C. auris isolates from the beach site were resistant to multiple antifungal drugs (Table S1). The susceptible and resistant isolates, when grown on agar plates containing serial dilutions of fluconazole, showed inhibition of growth of the susceptible isolate on the agar plate containing 16 mg/liter of fluconazole (Fig. 2). All of the C. auris isolates grew well at 42°C; however, the susceptible isolate (VPCI/E/AN/176/20) from the salt marsh grew slower than other isolates at both 37°C and 42°C. For example, isolate VPCI/E/AN/176/20 had a growth rate *r* of 0.212 and *r* of 0.117 in liquid media at 37°C and 42°C, respectively, which was lower than those of other isolates from site A (*n* = 1) and site B (*n* = 3) (37°C, *r* > 0.249, *P* = 0.03, versus 42°C, *r* > 0.143, *P* = 0.04). (Fig. S1). Further, isolate VPCI/E/AN/176/20 was highly susceptible to cycloheximide with 99% inhibition at 0.01 mg/liter whereas other C. auris isolates were inhibited only at a concentration of 2 to 4 mg/liter. Whole-genome sequencing (WGS) of 13 C. auris isolates including both isolates from site A and 11 from site B showed that they all clustered into clade I. However, they differed from all known south Asian clade I strains. Interestingly, while the Andaman isolates differed from the reference clade I strain B8441 from Pakistan by 829 to 904 single nucleotide polymorphisms (SNPs), they showed closer relationships to clinical strains from mainland India (Fig. 3). Interestingly, while the two isolates from site A were genetically different from each other by 77 SNP differences, all 11 isolates from site B were genetically very similar to each other, with no or 1 SNP difference across the whole genome, likely representing recent clonal descendants of a single ancestor genotype. Further, the two site A isolates differed from site B isolates by 77 to 78 SNPs.

10.1128/mBio.03181-20.1 FIG S1 The graph illustrates the growth curve (optical density versus time plot) of Candida auris strains isolated from the South Andaman district (SAD), Andaman & Nicobar Islands, Union Territory of India, at 37°C and 42°C. Download FIG S1, TIF file, 0.07 MB. Copyright © 2021 Arora et al. 2021 Arora et al. https://creativecommons.org/licenses/by/4.0/ This content is distributed under the terms of the Creative Commons Attribution 4.0 International license.

10.1128/mBio.03181-20.2 TABLE S1 MIC distribution of Candida auris (*n* = 24) isolated from the South Andaman district, Andaman & Nicobar Islands, Union Territory of India, against 8 antifungal drugs tested using the CLSI broth microdilution method. Download Table S1, DOC file, 0.03 MB. Copyright © 2021 Arora et al. 2021 Arora et al. https://creativecommons.org/licenses/by/4.0/ This content is distributed under the terms of the Creative Commons Attribution 4.0 International license.

For comparative analyses, we also included the WGS data of 18 strains from India published previously and nine clinical C. auris strains recently obtained from patients’ body sites and their hospital environment from the V. P. Chest Institute. A range of SNP differences were found. For example, there were 67 to 153 SNPs between our Andaman wetland strains and our nine hospital clinical strains. Similar genetic variability was noted among the Andaman isolates and four previously published C. auris strains (SNP differences, 46 to 126) from candidemia patients collected from 4 different hospitals of North and South India (14). Similarly, the Andaman isolates differed from 14 other Indian clade I isolates reported by Lockhart et al. (15) by 1 to 131 SNPs. Together, these results suggest that the Andaman isolates are genetically distinct but part of the broad C. auris population from India.

Our analyses identified that all 13 sequenced isolates were mating type a. Identification of genetic determinants of antifungal susceptibilities showed the presence of previously known amino acid substitution Y132F in the *ERG11* gene of all the 13 C. auris isolates. Interestingly, the *TAC1B* gene (a zinc-cluster transcription factor-encoding gene) had previously known amino acid substitution A583S in site A susceptible C. auris strain VPCI/E/AN/176/20. All C. auris isolates showed amino acid substitution L351M in the *ERG7* gene (ergosterol biosynthetic gene) and harbored the K719N amino acid substitution in the *STE6* gene, which is an ABC family transporter expressed in *MTLa*-carrying strains and exports the a-factor pheromone in Saccharomyces cerevisiae and Candida albicans.

### Discussion.

The present study reports the isolation of C. auris from the tropical marine ecosystems in India. The isolation of C. auris from this natural environment is noteworthy considering that until now this yeast has not been identified outside hospital environmental settings. Remarkably, C. auris was isolated from two different habitats, i.e., salt marsh wetland and sandy beach of the Andaman Islands, India. However, the ecological significance of the presence of C. auris in these habitats to human infections remains to be explored. Previously, *Candida* spp. of the Metschnikowia clade, namely, *C. haemulonii*, C. pseudointermedia, C. intermedia, C. melibiosica, and C. torresii, have been isolated from seawater and other coastal habitats from different geographical areas (16). The fact that many environmental isolations of *Candida* spp. of the Metschnikowia clade have occurred in tropical areas, predominantly in South and Southeast Asia, suggests potentially greater yeast diversity in tropical rather than temperate regions (17). The Andaman & Nicobar Islands have unique untouched ecology in terms of anthropogenic activities, and salt marsh and mangroves are the only wetland types in these islands that have vegetation. The isolation of C. auris from the salt marsh with extensive vegetation is consistent with the hypothesis that C. auris probably existed as a plant saprophyte in the wetlands (12). Notably, isolation of the drug-susceptible C. auris strain from this aquatic habitat with no known human activity probably indicates that C. auris existed as a drug-susceptible pathogen and developed multidrug-resistant traits after its adaptation in humans. Further, the drug-susceptible isolate grew slowly at high temperature, suggesting that environmental strains could acquire thermal tolerance quickly. The isolation of C. auris from the sediment of the nearshore environment may also be related to human activities and/or its close association with plants and soils in these environments. Regardless, the isolation of viable C. auris in both the marine habitats confirms that C. auris is capable of surviving harsh wetlands.

The environmental strains from the two sites had several genotypes and belonged to the South Asia clade I, similar to the C. auris clinical strains reported from India. Interestingly, Candida albicans was isolated from oak trees in an ancient wood pasture (18). Candida albicans is traditionally regarded as an obligate commensal of humans and other mammals, but the genomic sequences of the oak strains were closely related to those from humans. The high genetic diversity of C. albicans from old oaks shows that they can live in this environment for extended periods of time. Similarly, isolation of C. auris from the marine environment suggests wetlands as a niche for C. auris outside its human host. The exploration of further ecological niches will determine if new clades of C. auris exist in these natural habitats.

### Experimental procedures. (i) Sampling sites.

The Andaman & Nicobar group of islands (6°45′ N and 13°45′ N latitude and 92°12′ E and 93°57′ E longitude), Union Territory of India, are a chain of 572 islands/islets located in the southeastern Bay of Bengal at a distance of 1,200 km from mainland India with a coast line of 1,962 km. The islands are surrounded by the Andaman Sea to the east and the Bay of Bengal to the west. The Andaman group has 325 islands which cover an area of 6,170 km^2^. The temperature variation is slight, between 22°C and 30°C, and average relative humidity is 79%. Eight locations distributed on three islands detailed in Table 1 and Fig. 1 were investigated.

### (ii) Environmental sampling.

At each site, three replicate samples of sediments and seawaters were collected during low tides within a period of 1 month in the dry season (February to March 2020). The sediment samples were taken from a depth of 5 cm using a sterile plastic PVC corer of 5 cm in diameter and placed in sterile zip-lock pouches. The triplicate sediment samples collected from one site were then mixed, representing one composite sediment sample for each sampling site. For collection of the seawater, 50-ml sterile bottles were used and the seawater samples were maintained separately. The samples were transported to the laboratory at 4°C.

### (iii) Processing of environmental samples and identification of yeast isolates.

To isolate yeasts from these samples, 2 g of sediments (retaining the natural content of water) was suspended in 8 ml of 0.85% NaCl, vortexed, and allowed to settle. Four hundred microliters of the suspension was plated in triplicate on Sabouraud dextrose agar plates supplemented with chloramphenicol (50 mg/liter) and gentamicin (0.75 ml/liter) (SDA-CG) and incubated at 28°C up to 7 days. The seawater samples were filtered using sterile 0.45-μm membrane filters, and the membranes were plated on SDA-CG plates and incubated at 28°C up to 7 days. The physical-chemical parameters of soil and water samples such as salinity, temperature, and pH were recorded, and dissolved oxygen were obtained using the modified Winkler method (19).

The SDA-CG plates were examined periodically after 24 h, and yeast-like colonies were subcultured on CHROMagar *Candida* medium for preliminary identification. All yeast isolates were identified by matrix-assisted laser desorption ionization–time of flight mass spectrometry (MALDI-TOF MS) (Bruker Biotyper OC version 3.1; Daltonics, Bremen, Germany) using the ethanol-formic acid extraction method (20). Molecular identification of all isolates was performed by sequencing the internal transcribed spacer (ITS) regions and the D1D2 region of the ribosomal DNA (rDNA) as described previously (20). All C. auris isolates were screened for growth characteristics at 37°C and 42°C, and their ability to assimilate sugars and sensitivity to cycloheximide was determined as described previously (21). Antifungal susceptibility testing of C. auris was done using the broth microdilution method according to CLSI M27-A3 (13). Antifungals tested were fluconazole, voriconazole, posaconazole, isavuconazole, micafungin, anidulafungin, and amphotericin B.

### (iv) Spot assay on fluconazole-containing SDA plates.

To perform the spot assay, SDA plates were prepared containing 2-fold serial dilutions of fluconazole. Briefly, a freshly prepared stock solution of fluconazole in dimethyl sulfoxide (DMSO) was used to prepare fluconazole-containing agar dilution plates of 2-fold dilution from 0.25 mg/liter to 32 mg/liter. Inoculum was prepared by streaking the isolate on freshly prepared SDA plates (without antifungal) and incubated at 37°C for a period of 18 to 24 h. The colonies obtained from the overnight culture were directly suspended into sterile 0.85% saline, and optical density (OD) was adjusted to 0.1 (∼10^6^ cells/ml) at 625 nm. Five microliters each of the fluconazole-susceptible strain (VPCI/E/AN/176/20), three fluconazole-resistant strains (VPCI/E/AN/175/20, VPCI/E/AN/179/20, and VPCI/E/AN/180/20), and reference C. auris KCTC 17810 (clade II) were spot inoculated on the fluconazole-containing agar plates. The plates were left undisturbed to air dry under aseptic conditions and were incubated at 37°C for 24 h.

### (v) Growth profiling.

The growth profiles were studied for four C. auris strains, including the two C. auris strains from site A and two representative C. auris strains from site B. All four C. auris strains were first grown in Sabouraud dextrose (SD) broth overnight at 30°C. From the overnight culture, cells corresponding to an optical density of 0.1 at 625 nm (OD_625_) were inoculated into fresh SD broth in a microtiter plate. The plate was then incubated at 37°C and 42°C up to 72 h. The optical density was recorded in an Infinite 200 Pro (Tecan, Switzerland) microplate reader at regular intervals (2 h). The OD_625_ values versus time were plotted.

### (vi) Genome sequencing.

Candida auris was subjected to whole-genome sequencing using Illumina HiSeq 4000. Sequencing libraries were prepared as described elsewhere (22). For comparative analyses, we also included 18 previously published genomes of Indian C. auris strains (B11200, B11201, B11205 to B11207, B11209, B11210, B11212 to B11218, VPCI/510/P/14, VPCI/692/P/12, VPCI/550/P/14, and VPCI/479/P/13), 9 recent isolates from patients and their immediate environments admitted to V. P. Chest Institute (VPCI/82/P/20, VPCI/80/P/20, VPCI/81/P/20, VPCI/E/41C/20, VPCI/E/25/20, VPCI/E/17W/20, VPCI/E/7LP/20, VPCI/E/3/20, and VPCI/83/P/20), and reference clade I strain B8441 from Pakistan.

The genome-wise single nucleotide polymorphisms (SNPs) were identified using the NASP pipeline (Northern Arizona SNP Pipeline, http://tgennorth.github.io/NASP/). Reads were trimmed using Trimmomatic v0.39 (23) and aligned against the reference genome using BWA v0.7.17 (24). SNP sites were identified using GATK v2.7.4 (25). The SNP sites were filtered out if they were located in the repetitive regions of the reference genome, had a coverage lower than 10×, or had less than 90% variant allele calls. For the phylogenetic analysis, all the 1,154 identified SNP sites among the 41 strains were concatenated. The maximum likelihood tree was constructed using RAxML based on 1,000 bootstrap replicates and the ASC_GTRCAT nucleotide substitution model. The phylogeny was visualized using an online tool, iTOL.

### (vii) Data availability.

The genome sequences of all 13 Candida auris strains isolated in the present study from the South Andaman district, Andaman & Nicobar Islands, Union Territory of India, are accessible through BioProject number PRJNA679832.
