# Supplementary figures and images for: Environmental Isolation of Candida auris from the Coastal Wetlands of Andaman Islands, India
Source: mBio. 2021 Mar 16;12(2):e03181-20. doi: 10.1128/mBio.03181-20 (PMC8092279; doi:10.1128/mBio.03181-20)

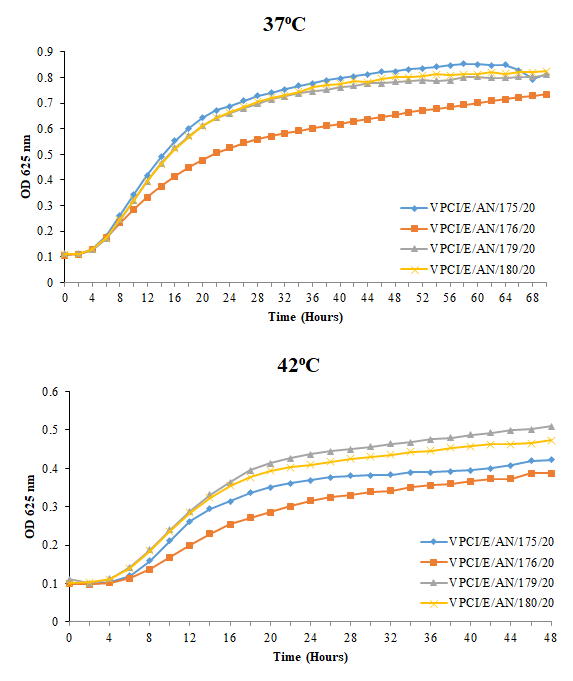

Supplement: FIG S1 [file mBio.03181-20-sf001.tif]
