# Supplementary material for: Environmental Isolation of Candida auris from the Coastal Wetlands of Andaman Islands, India
Source: mBio. 2021 Mar 16;12(2):e03181-20. doi: 10.1128/mBio.03181-20 (PMC8092279; doi:10.1128/mBio.03181-20)
Supplement: TABLE S1 [file mBio.03181-20-st001.doc]

**Table S1:** MIC distribution of *Candida auris* (n = 24) isolated from the South Andaman district (SAD), Andaman & Nicobar Islands, Union territory of India against 9 antifungal drugs tested using CLSI-BMD method.

| **Site (number of isolates)** | **Isolate ID** | **Parameters** | **MIC (mg/L)** | | | | | | | |
| --- | --- | --- | --- | --- | --- | --- | --- | --- | --- | --- |
|  |  |  | **FLU** | **ITC** | **VRC** | **ISA** | **POS** | **AMB** | **MFG** | **AFG** |
| ^#^A (n=2) | VPCI/E/AN/176/20 | MIC | 8 | 0.25 | 0.125 | <0.015 | 0.06 | 1 | 0.25 | 0.125 |
|  | VPCI/E/AN/175/20 | MIC | >256 | 1 | 2 | 0.5 | 0.25 | 2 | 0.125 | 0.125 |
| B (n= 22) | VPCI/E/AN/177/20-  VPCI/E/AN/198/20 | Range | 256->256 | 0.5-1 | 1-2 | 0.5 | 0.25 | 2-4 | 0.125-0.25 | 0.125 |
|  |  | GM MIC | 256 | 0.97 | 1.71 | 0.5 | 0.25 | 4 | 0.19 | 0.125 |
|  |  | MIC_50_ | 256 | 1 | 2 | 0.5 | 0.25 | 4 | 0.25 | 0.125 |
|  |  | MIC_90_ | 256 | 1 | 2 | 0.5 | 0.25 | 4 | 0.25 | 0.125 |

^#^Site A, Salt Marsh; Site B, Tourist Beach

MIC, Minimum Inhibitory Concentration; GM, Geometric Mean; MIC_50_, MIC at which 50% of test isolates were inhibited; MIC_90_, MIC at which 90% of test isolates were inhibited; FLU, Fluconazole; ITC, Itraconazole; VRC, Voriconazole; ISA, Isavuconazole; POS, Posaconazole; AMB, Amphotericin B; MFG, Micafungin; AFG, Anidulafungin.
